# The Emerging Challenges and Strengths of the National Health Services: A Physician Perspective

**DOI:** 10.7759/cureus.38617

**Published:** 2023-05-05

**Authors:** Zahid Khan

**Affiliations:** 1 Acute Medicine, Mid and South Essex NHS Foundation Trust, Southend-on-Sea, GBR; 2 Cardiology, Barts Heart Centre, London, GBR; 3 Cardiology and General Medicine, Barking, Havering And Redbridge University Hospitals NHS Trust, London, GBR; 4 Cardiology, Royal Free Hospital, London, GBR

**Keywords:** strengths of healthcare, challenges in healthcare, diversity and inclusion, covid – 19, medical staff, national health services, nhs approved medications, healthcare inequality, healthcare transition, global healthcare systems

## Abstract

The National Health Services (NHS) is a British national treasure and has been highly valued by the British public since its establishment in 1948. Like other healthcare organizations worldwide, the NHS has faced challenges over the last few decades and has survived most of these challenges. The main challenges faced by NHS historically have been staffing retention, bureaucracy, lack of digital technology, and obstacles to sharing data for patient healthcare. These have changed significantly as the major challenges faced by NHS currently are the aging population, the need for digitalization of services, lack of resources or funding, increasing number of patients with complicated health needs, staff retention, and primary healthcare issues, issues with staff morale, communication break down, backlog in-clinic appointments and procedures worsened by COVID 19 pandemic. A key concept of NHS is equal and free healthcare at the point of need to everyone and anyone who needs it during an emergency. The NHS has looked after its patients with long-term illnesses better than most other healthcare organizations worldwide and has a very diversified workforce. COVID-19 also allowed NHS to adopt newer technology, resulting in adapting telecommunication and remote clinic.

On the other hand, COVID-19 has pushed the NHS into a serious staffing crisis, backlog, and delay in patient care. This has been made worse by serious underfunding the coronavirus disease-19coronavirus disease-19 over the past decade or more. This is made worse by the current inflation and stagnation of salaries resulting in the migration of a lot of junior and senior staff overseas, and all this has badly hammered staff morale. The NHS has survived various challenges in the past; however, it remains to be seen if it can overcome the current challenges.

## Editorial

Healthcare systems worldwide have been under immense pressure due to increased demand, staffing issues, and an aging population [1]. The COVID-19 pandemic has highlighted several key aspects of NHS, including its resilience, cultural diversity, and reliability [1]. It has also exposed the weakness within the system, such as workforce shortages, increasing backlog of care and appointments, delay in providing care to patients with even emergency care, and serious illnesses such as cancer [2]. The NHS has seen various up and downs since its creation in 1948, but COVID-19 and significant underfunding over the last decade threaten its existence.

Strengths

The strengths of NHS include its workforce, who have gone above and beyond during the pandemic to support patients and relatives. Their selflessness and commitment have been amazing, and they have put their lives and licenses at risk by going the extra mile to help patients and families in resource-deprived systems [1]. The second strength of the NHS is that it is a public-funded national health service and has strong central leadership. Public support for NHS remains high despite the enormous challenges it is facing [2]. Staff diversity is another key strength of the NHS which is partly due to its international recruitment, and the United Kingdom's (UK) recruitment of medical and nursing staff remains one of the highest in the world. The NHS Wales recruited over 400 nurses from overseas last year, and this number is likely to rise due to an increase in demand and lack of supply in the local market [3]. The Medical Workforce Race Equality Standard (MWRES) reported an increase of 9000 doctors from BAME backgrounds in the NHS, increasing from 44,000 to 53,000 since 2017 [4]. This equals 42% of medical staff working in the NHS now coming from BAME backgrounds. Although BAME doctors remain underrepresented in senior positions, this number is increasing, and the number of medical directors from BAME backgrounds increased to 20.3% in 2021 [4]. The NHS is a centrally funded healthcare that is free at the point of delivery, although over the last few years, a health surcharge has been introduced for visitors from overseas and migrants working in the UK on tier 2 visas. Another key strength of the NHS is public satisfaction which remains high despite the various challenges and shortcomings faced by the NHS [5]. The productivity of the NHS has increased over time, although measuring true productivity can be difficult. A study by the University of York's Centre for Health Economics found that the average annual NHS productivity growth was 1.3% between 2004-2017, and the overall productivity increased by 416.5% compared to 6.7% productivity growth in the economy. Based on the Commonwealth Fund analysis, the NHS comes 4th out of 11 systems and compares well with other healthcare systems [4,6]. Traditionally, NHS has been very slow to accept digital technology for various reasons, but since the COVID-19 pandemic, this has changed, and there is increasing use of technology such as video and telephonic appointments. This is likely to increase further and will prove cost-effective in the long run.

Challenges

**Figure 1:**
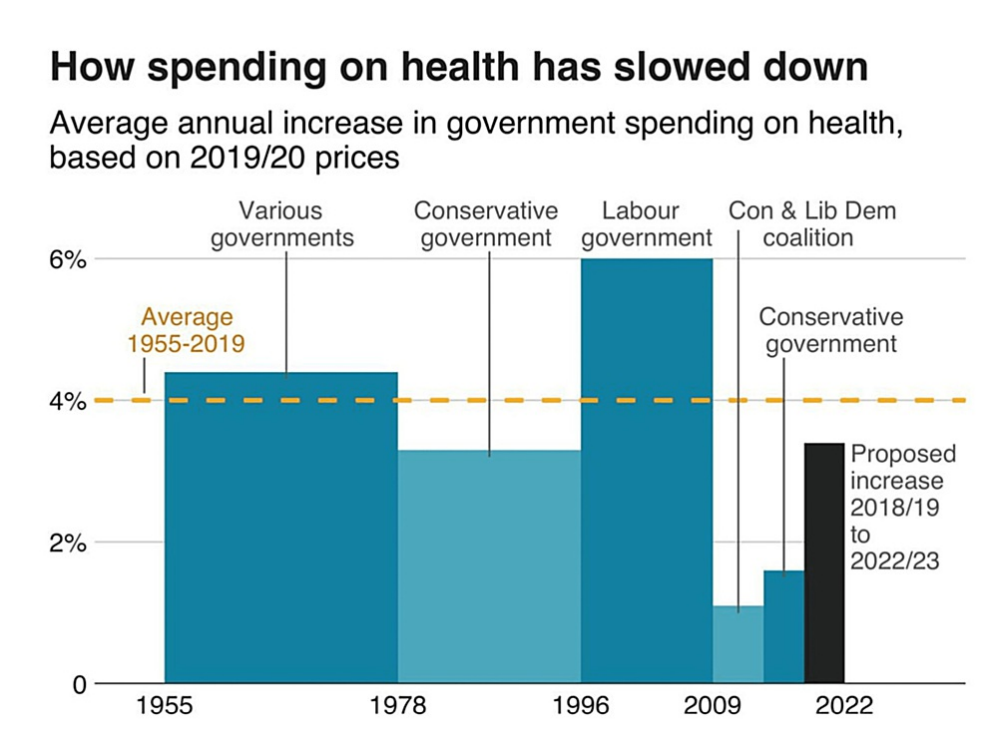
The NHS spending summary National Health Services (NHS) [3] Permission obtained from the authors

There are several challenges faced by the NHS, ranging from staff shortages, retention, financial issues, patients care backlog, healthcare inequalities, social care issues, and evolving healthcare needs. COVID-19 affected ethnic minority communities, and people from poor areas more than others, and the UK life expectancy has fallen recently compared to other European countries [3]. The hospital bed crisis during the pandemic was mainly due to excessive underfunding of the NHS, and it resulted in a significant number of failings for patients, relatives, and service providers, and deaths. The social care system needs urgent attention and funding [4]. The annual spending on NHS increased by 4% every year; however, this number has dropped to 1.5% since the 2008 financial crisis, which is well below the average annual spending [5]. Although the government planned an increase in this spending to 3.4% for the next few years from 2019-20, the rising inflation and pandemic mean that this spending is still far below the average annual spending of NHS (Figure 1).

**Figure 2:**
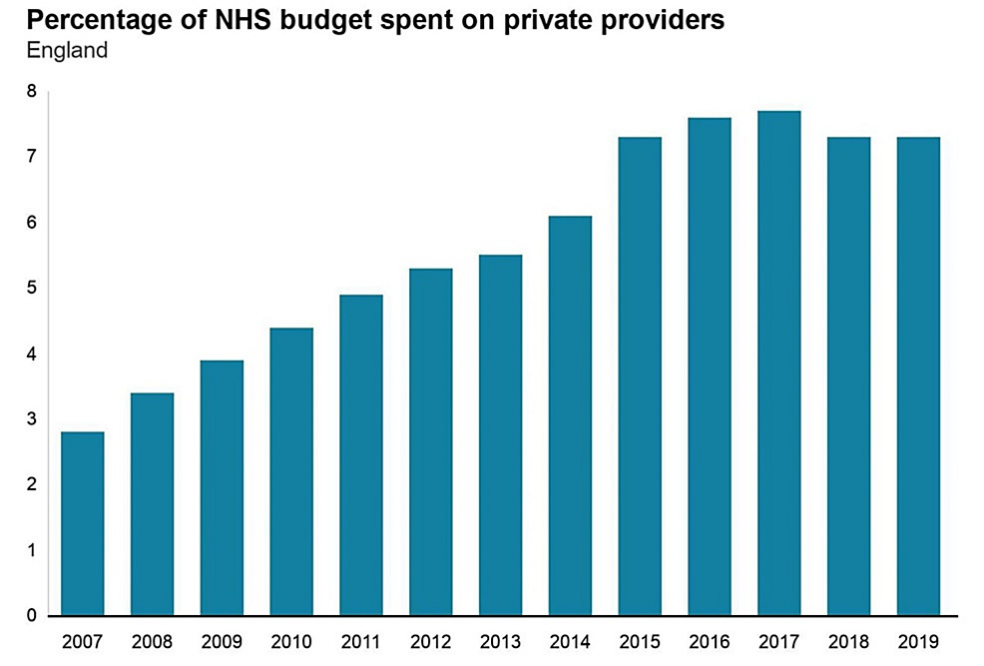
The Health and Social care department report on the participation of private companies in NHS The National Health Services (NHS) [3]. Permission obtained from the authors

Due to years of poor workforce planning, weak policies, and fragmented responsibilities, there is a serious staffing crisis in both health and social care. This has been made worse by constant pay erosion for staff and workforce unfriendly pension policies resulting in a significant number of healthcare and social care staff retiring or moving abroad in search of better work-life balance and better pay. The latest junior doctors and nursing strikes are a clear example of that. NHS offered more primary care appointments to patients last year compared to the pre-pandemic level despite a falling number of general practitioners. There are also inequalities in academia due to hierarchical structures and precarious roles held disproportionately by women and UK ethnic minorities [5]. The annual report by Health and Social care department highlighted the increasing privatization of the NHS, and more private companies had taken control of its services, as shown in Figure 2.

**Figure 3:**
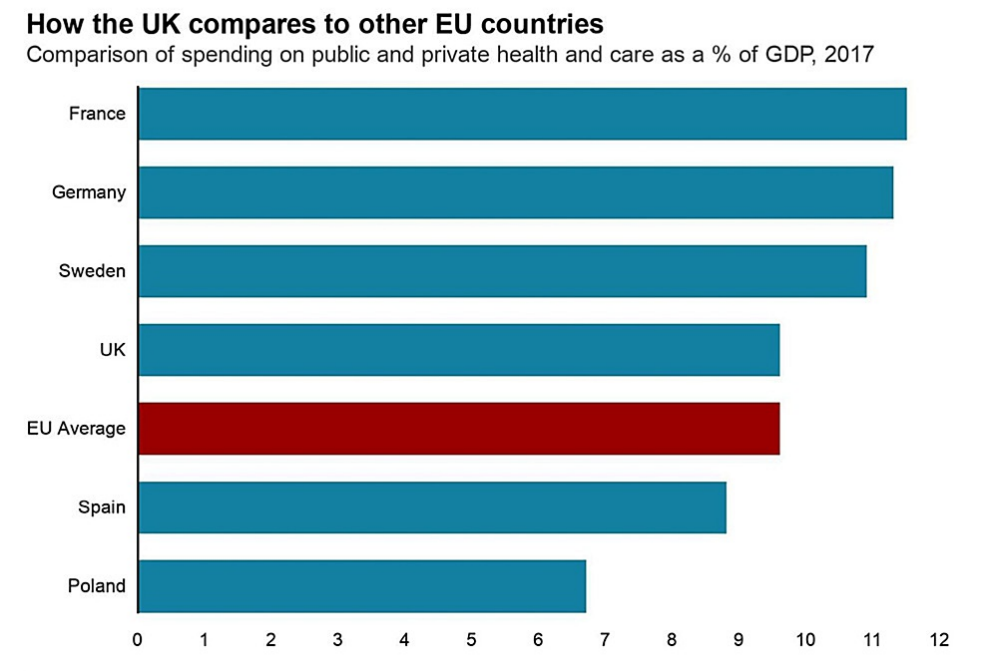
The percentage of gross domestic product comparison between the UK and other European countries. United Kingdom (UK) [3] Permission obtained from the authors

The aging population is another key challenge faced by the NHS which is not only due to a significant number of complicated health issues but also social care need. A significant increase in NHS spending on social care is required to overcome this issue. The recent data shows that, on average, an ill 65-year-old patient costs NHS 2.5 times more than a 30-year-old. The proportion of GDP spent by the UK on the NHS is less compared to other European countries, and this figure has got worse over the past decade (figure 3). The NHS is unlikely to cope with the major challenges it is facing without a significant increase in social and healthcare spending [3].

The number of medical and non-medical staffing vacancies remains very high in the NHS. This is partly made worse by the current pension issues and pay cuts for medical and non-medical staff, which has forced them to abandon healthcare or move overseas. Despite the government plan to increase the number of medical school placements over the years, this is unlikely to solve the problem due to the lack of a retention plan. For example, the UK government increased the number of medical school placements from 6000 to 7500 in 2018, but this is unlikely to solve the problem as these new graduates start thinking about going overseas or taking gap years due to the enormous amount of pressure, they are under during training period [6].

Recommendations and interventions

It is time for certain steps to be taken to address these key challenges. For example, it is unlikely to retain healthcare staff without offering attractive pay offers, opportunities for flexible working, and clearer career pathways. Staff well-being should be at the heart of NHS reformation, and they should be given time, space, and resources to recover to deliver the best possible care to their patients. The British Medical Association (BMA) made a number of proposals to the UK government regarding the pension scheme, such as rolling out of recycling of unused employer contributions more widely and can be passed onto opted-out members of the pension scheme, although this approach has its own limitations. Additionally, the lifetime pot threshold needs to be increased to retain health staff. In addition, the government should allow pension growth across both the NHS pension scheme and the reformed scheme to be aggregated before testing it against the annual allowance [7,8]. The current industrial action by NHS nurses and junior doctors and consideration of similar steps by the consultant body of the BMA perhaps should be an eye opener for the looming NHS staffing crisis. This can be best tackled by the government negotiating with the unions in a flexible way and offering them a reasonable pay rise that accounts for the pay deduction they have encountered since 2007. The four UK nations have shown divergence of opinion and recommendations on tackling this issue as NHS Scotland has agreed with NHS staff, but the crisis seems to be worsening in NHS England.

More must be done to tackle racism and discrimination within the NHS and equal opportunities should be provided to minority healthcare and social care workers. This can be done in several ways, but the most important step is acknowledging that this exists in the first place. All staff members should be provided training to recognize racism and empower them to take actions to tackle racism within the workplace. Similarly, steps should be taken to create equal opportunities for staff from the BAME community for career progression and development. Organizations need to demonstrate that they are willing to make the difficult decision of allowing staff members to have a conversation about racism without fear of repercussions. The NHS has developed tools to report racism witnessed or experienced at the workplace, but more needs to be done, and putting cultural safeguards would be a reasonable step. Organizations can arrange cultural events for staff to have meaningful conversations about anti-racism policies put in place to highlight areas of improvement [6]

There is a need at the leadership level to develop and show compassion to the front-line staff. The government needs to take steps and create policies to tackle the inequalities laid bare by the pandemic. A significant number of deaths in care homes during the COVID-19 pandemic showed that the social care setup is not fit for purpose and needs reformation on an urgent basis. This can only be addressed by increasing funding, better pay, and working conditions for the social care workforce. The NHS needs investment in building a digital infrastructure and tools, and public health and care staff must be involved in this process [9]. The NHS public funding has increased from 3.5% in 1950 to 7.3% in 2017, but this is not enough to keep up with the inflation and other issues faced by NHS [10]. Borrowing more money for the NHS is only a short term solution and to fund the NHS properly, the government might need to increase taxes on all households. Although the public generally will agree to higher taxes to fund the NHS, this might prove difficult with rising inflation and increasing poverty. Another option could be to divert funding from other areas to the NHS, but this will affect the development being made in other sectors. A recent survey of the British public showed that they are willing to pay higher taxes provided the money was spent on NHS only, and this perhaps needs more accountability to avoid wasting NHS money [10].
